# Establishing an objective clinical spectrum, genotype-phenotype correlations, and *CRMP1* as a modifier in the Ellis-van Creveld syndrome: The first systematic review of *EVC-* and *EVC2*-associated conditions

**DOI:** 10.1016/j.gimo.2023.100781

**Published:** 2023-03-13

**Authors:** Jorge Diogo Da Silva, Ana Rita Soares, Ana Maria Fortuna, Nataliya Tkachenko

**Affiliations:** 1 Centro de Genética Médica Doutor Jacinto Magalhães, Centro Hospitalar Universitário de Santo António, Porto, Portugal; 2 Life and Health Sciences Research Institute (ICVS), School of Medicine, University of Minho, Braga, Portugal; 3 ICVS/3B’s – PT Government Associate Laboratory, Braga/Guimarães, Portugal; 4 Unit for Multidisciplinary Research in Biomedicine, Abel Salazar Biomedical Sciences Institute, Porto University, Porto, Portugal

**Keywords:** *CRMP1*, Ellis-van Creveld, *EVC*, *EVC2*, Skeletal ciliopathy

## Abstract

**Purpose:**

Ellis-van Creveld (EVC) syndrome is an autosomal recessive skeletal ciliopathy that was first identified in the Old Order Amish. Since its discovery, two causal genes have been identified, *EVC* and *EVC2*, showing that several cases were misdiagnosed and were, in fact, other entities. Nevertheless, there has not been any adequate phenotypic characterization of molecularly defined EVC syndrome so far.

**Methods:**

We performed a systematic review of case reports of EVC syndrome with molecular confirmation of pathogenic variants in *EVC* or *EVC2*. Demographic, genetic, and clinical information of patients was assessed.

**Results:**

We reviewed 725 papers and obtained 54 case reports/series that met the inclusion criteria, with a total subject sample of 310. Of these, 190 had biallelic variants, whereas 28 were affected heterozygotes. Our analysis revealed new phenotypes that have not been classically linked to the syndrome and others that have been linked but are very rare. Monoallelic symptomatic forms had less expressivity, and biallelic cases were milder if associated with *EVC* and/or missense variants. Finally, we identified *CRMP1*, a gene whose coding region partially overlaps with *EVC*, as a potential genetic modifier of the severity of the EVC syndrome.

**Conclusion:**

We provided the first objective clinical characterization of molecularly defined EVC syndrome and identified the first associated genetic modifier, *CRMP1*, which had not been implicated in human disease before.

## Introduction

Ellis-van Creveld (EVC) syndrome is a skeletal ciliopathy, which was clinically described in 1963 in the Old Order Amish. McKusick et al^1^ described both males and females presenting with short-limbed disproportionate dwarfism, polydactyly, dysplasia of fingernails, tight fists, fusion of the hamate and capitate bones of the wrist, and knocked-knees. The analysis of extended pedigrees led to the conclusion of an autosomal recessive inheritance for this syndrome.^2^^,^^3^ Since then, several studies and reports have been describing patients and symptoms associated with EVC syndrome. Other described features include cardiac anomalies (mostly atrial septal defects), short ribs, cone-shaped epiphyses, teeth anomalies, male genitalia malformations, and mild intellectual disability.^4^, ^5^, ^6^, ^7^, ^8^, ^9^, ^10^ Prenatal cases have been described, too.^11^, ^12^, ^13^, ^14^, ^15^ Furthermore, an apparently milder form of the condition, associated with autosomal dominant inheritance, was also described and named Weyers acrofacial dysostosis (WAD).^16^ However, because the diagnosis was clinical, many different entities would easily be mistaken by EVC syndrome. This scenario only started changing with the discovery of the genes responsible for the syndrome: *EVC* in 2000 and *EVC2* in 2002.^17^, ^18^, ^19^, ^20^, ^21^, ^22^ The EVC syndrome and WAD were found to be caused by biallelic and monoallelic *EVC*/*EVC2* variants, respectively.^23^^,^^24^ At this time, about one-third of cases of previously diagnosed EVC syndrome were not associated with *EVC* or *EVC2* and were therefore misdiagnosed. Therefore, the molecular confirmation became an essential step for the correct diagnosis and genetic counselling.

*EVC* and *EVC2* are located on 4p16.2 chromosomal region, with divergent orientation and separated by 2.6 kb of genomic sequence. *EVC* has 21 coding exons spanning 103 kb of genomic DNA, which translate into a 992 amino acid protein. This gene is expressed in the developing skeleton (mainly vertebral bodies, ribs, and both upper and lower distal limbs), heart (both atrial and interventricular septa), kidney, and lung. *EVC2* has 22 coding exons spanning 166 kb of genomic DNA, encoding a 1308 amino acid protein with a similar expression pattern to *EVC*.^17^^,^^19^

EVC and EVC2 are N-terminal–anchored membrane proteins, which are complexed together in the basal body of primary cilia, a crucial signal-transducing structure for development.^25^^,^^26^ These proteins are mutually required for their localization in primary cilia and have an essential function in hedgehog (Hh)-mediated signaling.^27^ Mouse and in vitro studies have showed that, during development, Hh binds to Protein patched-1, inducing its removal from primary cilia, which allows the release of the constitutively repressed Smoothened (Smo) protein.^28^ Smo then functions as a transcriptional activator, leading to the production of different Gli proteins and Sufu.^29^ Smo also interacts with the Evc/Evc2 complex, therefore allowing both translocation of Gli proteins and Sufu to the tip of the primary cilia and dissociation of Sufu/Gli3 complexes.^27^ The Gli proteins are activated throughout the intraflagellar transport system and ultimately modulate proliferation and differentiation of developing tissues.^27^^,^^30^ Disruption of Evc or Evc2 impairs the effect of Smo in the production/activation of Gli proteins, affecting the development of different tissues through different mechanisms, not all of which are fully clear.^27^ Nevertheless, Hh signaling is required for normal development of the orofacial region (including of the teeth), the endochondral growth plate of the axial skeleton, and for respiratory endoderm patterning for normal cardiac morphogenesis.^31^ One of the well-established pathological mechanisms associated with Evc/Evc2 dysfunction in the perichondrium is the loss of the fibroblast growth factor–inhibiting role of Hh signaling.^32^ This leads to unopposed fibroblast growth factor–dependent inhibition of chondrocyte proliferation and, consequently, skeletal underdevelopment.^32^ Overall, EVC and EVC2 are key players of a complex signaling process, which is required for adequate development of several endodermal and ectodermal structures.

Although there are many articles in the literature describing several variants or clustering of signs/symptoms, by the time of this project, to our knowledge, there were no articles merging the clinical characterization of patients with confirmed molecular diagnosis or establishing genotype-phenotype correlations regarding the affected gene, type of variant, and allelism. Characterizing the specific gene-associated clinical condition is an essential step to find other previously unreported signs or symptoms, or to exclude previous incorrectly associated ones. Moreover, this characterization could lead to a better understanding of the manifestation in different stages of life (including the prenatal setting) and to predict the penetrance and variable expression of monoallelic variants. Hence, we performed the first systematic review of all published cases of EVC syndrome with confirmed molecular diagnosis to address this gap.

## Materials and Methods

### Systematic review approach

The systematic review was performed in accordance with the COSMOS-E (Conducting Systematic Reviews and Meta-Analyses of Observational Studies of Etiology) guidelines^33^ where applicable for a systematic review of case reports. Reporting of the results of the systematic review was performed in accordance with the PRISMA (Preferred Reporting Items for Systematic Reviews and Meta-Analyses) 2020 statement.^34^

We performed a literature search on PubMed® using the following query: “(Ellis-van Creveld Syndrome) OR (EVC protein or human) OR (EVC2 protein or human) OR ((*EVC* gene) AND (human)) OR ((*EVC2* gene) AND (human)) OR (Weyers acrofacial dysostosis) OR (Weyers acrodental dysostosis)”. Results up to August 20, 2022, were included in the analysis.

Additionally, we assessed all relevant genetic variants for the *EVC* and *EVC2* gene in the ClinVar® database. We performed a search with the queries “evc[gene]” and “evc2[gene]” (HGNC:3497 and HGNC:19747, respectively). For each of the results, we applied the following filters: “< 1kb, single gene,” “> 1kb, single gene,” “Likely pathogenic,” and “Pathogenic.” For each gene, all individual variants were assessed for associated literature references. We found that all the cited references were included in the results of our literature search; therefore, no additional reference was added to the pool of studies to evaluate. Finally, we included 2 original cases from our center, which were previously unpublished.

### Result filtering

Inclusion criteria for each individual study were as follows: (1) publication of a case report or case series with a clinical phenotype description and (2) molecular confirmation of a likely pathogenic or pathogenic variant in the *EVC* or *EVC2* gene. Reports with clinical description in which molecular confirmation was performed in a subsequent publication were also included. Exclusion criteria were as follows: (1) case reports in nonhuman species; (2) insufficient or absent clinical description of a case with molecular diagnosis; (3) absent, incomplete, or inconclusive molecular confirmation; (4) and molecular diagnosis in genes other than *EVC* or *EVC2*. All manuscripts in English, Spanish, French, Italian, or Portuguese were considered for appraisal.

**Figure 1:**
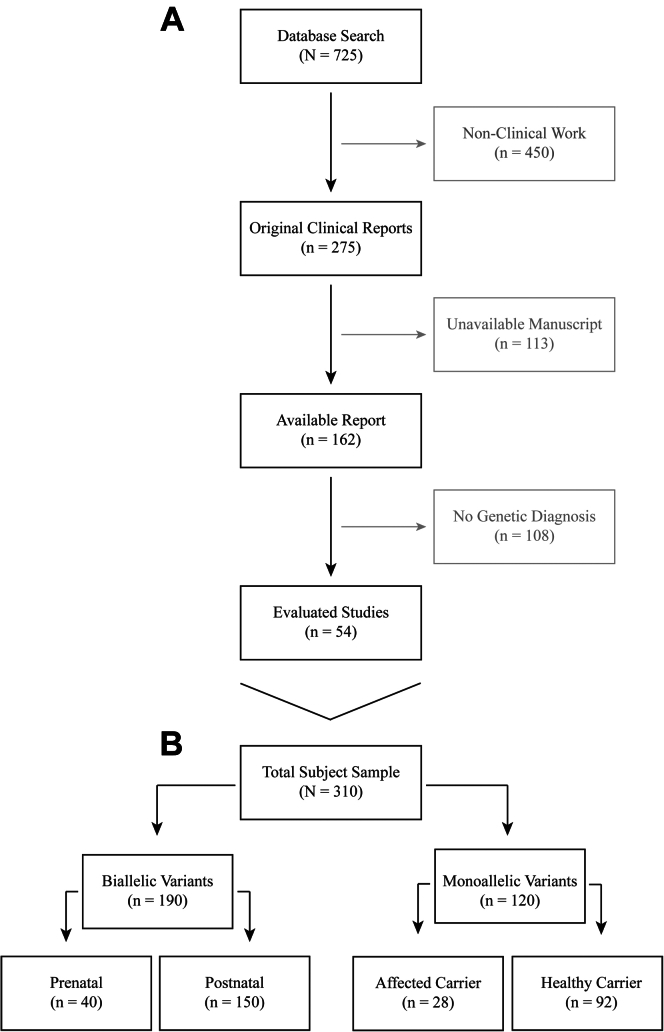
**Flowthrough of the systematic review and collected sample.** (A) Flowthrough of the systematic review, with indication of excluding criteria for assessed manuscripts. The sample size refers to the number of published manuscripts. (B) Schematic of the patient sample, with sample size referring to the number of individual cases (independently of being in the same family or not).

Assessment of the total number of obtain reports was carried out by 2 researchers in parallel. Each research performed an independent evaluation of each manuscript according to the flowthrough in Figure 1A. The initial search obtained a total of 725 reports, published between 1951 and 2022. We excluded 450 manuscripts of nonclinical reports, 113 manuscripts that were unavailable online (usually older reports without indication of a genetic diagnosis or published before the identification of the causal genes) or in a nonincluded language, and 108 manuscripts with clinical cases without a molecular diagnosis. A final number of 54 studies was obtained for inclusion in this study.

### Data processing and statistical analysis

Each individual patient comprised in the final pool of reports was considered as an individual datapoint. Clinical cases were assessed for phenotypes that are reported in the OMIM (Online Mendelian Inheritance in Man) database for the Ellis-van Creveld syndrome (MIM 225500) and for Weyers acrofacial dysostosis (MIM 193530). Additional features not reported in the OMIM clinical synopsis of either condition were also registered in the database. We also registered demographic information, if available, as well as the genetic variants and allelism.

Data processing and analysis was performed using SPSS 26.0 (IBM Corp.) and GraphPad Prism version 9.3.0 for Windows (GraphPad Software). Data regarding phenotype frequency are presented by their relative proportion to the total number of subjects in which that variable was assessed and reported. Statistical comparison of proportions was performed using the χ^2^ test, assuming a 95% confidence interval, and using the Cramer’s V value as an effect size measure. Effect size cutoffs for Cramer’s V were 0.100, 0.300, and 0.500 for a small, medium, and large effect, respectively. For the comparison of phenotype frequency between different types of variants, individual variants in one allele were considered, and the sample size is therefore virtually duplicated: to correct for this, the χ^2^ test was adjusted for half the sample size of each variant type.

## Results

From the 54 eligible studies, a total of 310 individual patients were described (Figure 1B). Of those, 190 (61%) had biallelic variants and 120 (39%) had monoallelic variants in either *EVC* or *EVC2*. All subjects with biallelic variants were affected. Of those with monoallelic variants, 28 (23%) were affected, and 92 (77%) were healthy heterozygotes.

### Phenotype characterization of subjects with biallelic variants

Demographic and genetic characteristics of subjects with biallelic variants are represented in Table 1. Sex distribution is balanced, and most cases are diagnosed in the postnatal setting, specifically, in the pediatric age group. More than half the patients have positive family history, and about two-thirds have consanguinity, which is a similar frequency to that of homozygous pathogenic variants. The *EVC* gene is slightly more commonly implicated, and there are cases of copy number variants (CNVs) that affect both *EVC* and *EVC2*. However, there is no reported case of digenic inheritance. Regarding diagnostic tests, variants were mostly detected by targeted Sanger sequencing, which likely reflects the number of cases that were diagnosed before the advent of next-generation sequencing.

**Table 1:** Demographic and genetic characterization of affected subjects

Variable	Biallelic *N* (%)^a^	Monoallelic *N* (%)^a^
Sex		
Male	94 (51.4%)	16 (57.1%)
Female	89 (48.6%)	12 (42.9%)
Age group		
Prenatal	40 (21.1%)	4 (14.3%)
Postnatal	150 (78.9%)	24 (85.7%)
Pediatric	103 (68.7%)	11 (39.3%)
Adult	26 (17.3%)	13 (46.4%)
Unknown	21 (14.0%)	0
Ethnicity		
African	8 (4.4%)	0
Arab	28 (15.3%)	0
Asian	30 (16.4%)	9 (32.1%)
Hispanic	17 (9.3%)	0
White	100 (54.6%)	19 (67.9%)
Positive family history	84 (56.8%)	24 (54.2%)
Consanguinity	124 (66.7%)	2 (7.4%)
Affected gene		
*EVC*	106 (55.8%)	4 (14.3%)
*EVC2*	77 (40.5%)	23 (82.1%)
Both^b^	7 (3.7%)	1 (3.6%)
Allelism		
Homozygous	132 (69.5%)	NA
Compound heterozygous	58 (30.5%)	
Variant type		
Missense	43 (11.3%)	5 (17.9%)
Nonsense	84 (22.1%)	9 (32.1%)
Frameshift	93 (24.5%)	12 (42.9%)
Splicing	109 (28.4%)	1 (3.6%)
CNV	52 (13.7%)	1 (3.6%)
Variant combination		
Missense Missense	17 (9.0%)	NA
Missense Truncating	9 (4.7%)	
Truncating Truncating	164 (86.3%)	
Diagnostic test		
Sanger (targeted)	125 (66.2%)	21 (75.0%)
NGS panel	31 (16.4%)	4 (14.3%)
ES/GS	14 (7.4%)	2 (7.1%)
Array	8 (4.2%)	1 (3.6%)
MLPA	4 (2.1%)	0
Multiple	7 (3.7%)	0

*CNV*, copy number variant; *EVC*, Ellis-van Creveld; *MLPA*, multiplex ligation-dependent probe amplification*;NA*, not applicable; *NGS*, next-generation sequencing; *ES*, exome sequencing; *GS*, genome sequencing.

a Phenotype frequency is represented by the absolute number (*N*) and respective proportion (%).

b Cases with “both” affected genes have either 1 or 2 copy number variants that affect both *EVC* and *EVC2* in cis, as there is no case with digenic inheritance.

General and specific clinical phenotypes (with a frequency above 5%) of subjects with biallelic variants are represented in Tables 2 and 3, respectively. As expected, skeletal anomalies were present in almost every patient, with bilateral postaxial polydactyly of the hands being the most common phenotype (present in 98% of subjects; vs 28% of postaxial polydactyly of the feet). The top phenotypic manifestations in frequency are related to skeletal changes or ectodermal dysplasia. Congenital heart defects were present in approximately 64% of subjects, atrial septal defect being the most common one. Genital symptoms were very uncommon, with no patient reporting cryptorchidism. Finally, various phenotypic manifestations not included in the OMIM were also common, namely, brachydactyly, syndactyly, short broad nose, and hypertelorism.

**Table 2:** General phenotypes (by system) in patients with biallelic and monoallelic variants

Feature	Biallelic % (*N*/Total)^a^	Monoallelic % (*N*/Total)^a^	χ^2^	Significance (*P*)	Cramer’s V
Skeletal anomaly	99.5% (186/187)	85.7% (24/28)	20.387	<.001^b^	0.307
Skeletal anomaly (excluding polydactyly)	89.7% (166/185)	78.6% (22/28)	2.923	.087	0.117
Facial feature	81.3% (139/171)	67.9% (19/28)	2.653	.103	0.115
Congenital heart disease	64.5% (109/169)	25.0% (7/28)	15.477	<.001^b^	0.280
Thoracic anomaly	58.0% (98/169)	14.3% (4/28)	18.374	<.001^b^	0.305
Neurological disease	6.0% (8/133)	10.7% (3/28)	0.802	.370	0.071
Genital anomaly	6.0% (5/83)	5.9% (1/17)	0.001	.982	0.002

a Phenotype frequency is represented by the absolute number (*N*) for the patients in which the phenotype was assessed (Total), with the respective proportion (%).

b *P* < .050.

**Table 3:** Specific phenotypes in patients with biallelic and monoallelic variants

Feature	Biallelic % (*N*/Total)^a^	Monoallelic % (*N*/Total)^a^	χ^2^	Significance (*P*)	Cramer’s V
Postaxial polydactyly (hands)	98.9% (186/188)	71.4% (20/28)	41.765	<.001^b^	0.440
Bilateral	98.9% (184/186)	90.0% (18/20)	7.554	.006^b^	0.191
Short stature	78.7% (74/94)	17.4% (4/23)	31.279	<.001^b^	0.517
Limb shortening	78.6% (143/182)	17.9% (5/28)	42.991	<.001^b^	0.452
Nail dysplasia/hypoplasia	72.0% (108/150)	63.0% (17/27)	0.901	.343	0.071
Hypodontia	66.0% (68/103)	60.9% (14/23)	0.219	.639	0.042
Alveolar ridge defect	60.4% (87/144)	25.9% (7/27)	10.927	.001^b^	0.253
Atrial septal defect	54.8% (91/166)	14.3% (4/28)	15.752	<.001^b^	0.285
Narrow chest	54.4% (92/169)	14.3% (4/28)	15.500	<.001^b^	0.281
Short ribs	53.3% (89/167)	14.3% (4/28)	14.626	<.001^b^	0.274
Short and thickened tubular bones	50.3% (75/149)	14.3% (4/28)	12.396	<.001^b^	0.265
Delayed eruption of teeth	48.9% (45/92)	60.0% (12/20)	0.808	.369	0.085
Other dysmorphisms	35.9% (55/153)	10.7% (3/28)	6.921	.009^b^	0.196
Other congenital heart defects	35.9% (60/167)	21.4% (6/28)	2.252	.133	0.107
Limb shortening at birth	35.8% (24/67)	0.0% (0/20)	9.893	.002^b^	0.337
Low iliac wings	31.7% (44/139)	16.7% (4/24)	2.213	.137	0.117
Low weight	29.8% (17/57)	0.0% (0/20)	7.655	.006^b^	0.315
Acetabula spur projections	28.1% (39/139)	16.7% (4/24)	1.367	.242	0.092
Postaxial polydactyly (feet)	27.6% (50/181)	60.7% (17/28)	12.190	<.001^b^	0.242
Bilateral	88.0% (44/50)	100.0% (17/17)	2.241	.134	0.183
Prenatal limb shortening	27.1% (19/70)	0.0% (0/20)	6.881	.009^b^	0.277
Upper-lip defect	26.5% (43/162)	0.0% (0/28)	9.606	.002^b^	0.225
Genu valgum	23.0% (38/165)	0.0% (0/28)	8.029	.005^b^	0.204
Abnormal birth stature	22.0% (13/59)	0.0% (0/20)	5.275	.022^b^	0.258
Brachydactyly	21.6% (36/167)	7.1% (2/28)	3.175	.075	0.128
Neonatal teeth	21.1% (19/90)	8.3% (2/24)	2.059	.151	0.134
Short broad nose	19.3% (29/150)	3.6% (1/28)	4.183	.041^b^	0.153
Ventricular septal defect	19.6% (31/158)	10.7% (3/28)	1.263	.261	0.082
Cone-shaped epiphyses of phalanges	18.4% (27/147)	17.9% (5/28)	0.004	.949	0.005
Syndactyly	16.2% (27/167)	42.9% (12/28)	10.676	.001^b^	0.234
Capitate-hamate fusion	16.3% (24/147)	0.0% (0/28)	5.298	.021^b^	0.174
Long philtrum	15.3% (23/150)	3.6% (1/28)	2.798	.094	0.125
Cleft lip	10.6% (17/160)	0.0% (0/27)	3.156	.076	0.130
Single atrium	10.2% (17/166)	7.1% (2/28)	0.260	.610	0.037
Low birth weight	8.8% (5/57)	5.0% (1/20)	0.293	.588	0.062
Postnatal microcephaly	8.8% (5/58)	0.0% (0/20)	1.876	.171	0.156
Developmental delay	8.8% (7/80)	12.5% (3/24)	0.299	.585	0.054
Prenatal microcephaly	8.0% (4/50)	0.0% (0/20)	1.697	.193	0.156
Pectus carinatum	7.5% (8/106)	0.0% (0/24)	1.930	.165	0.122
Clinodactyly	7.2% (12/167)	7.1% (2/28)	< 0.001	.994	0.001
Hypertelorism	6.0% (9/150)	3.6% (1/28)	0.262	.608	0.038
Club foot	5.1% (8/157)	3.6% (1/28)	0.119	.730	0.025

a Phenotype frequency is represented by the absolute number (*N*) for the patients in which the phenotype was assessed (Total), with the respective proportion (%).

b *P* < .050.

### Comparison of patients with biallelic vs monoallelic variants

Regarding symptomatic subjects with monoallelic variants (Table 1), which were much less frequent than those carrying biallelic variants, positive family history was present in just more than 50% of cases, similar to biallelic forms, whereas consanguinity was much less frequent (present in 7.4% of cases). Most monoallelic variants affected *EVC2* and were truncating (Table 1).

**Figure 2:**
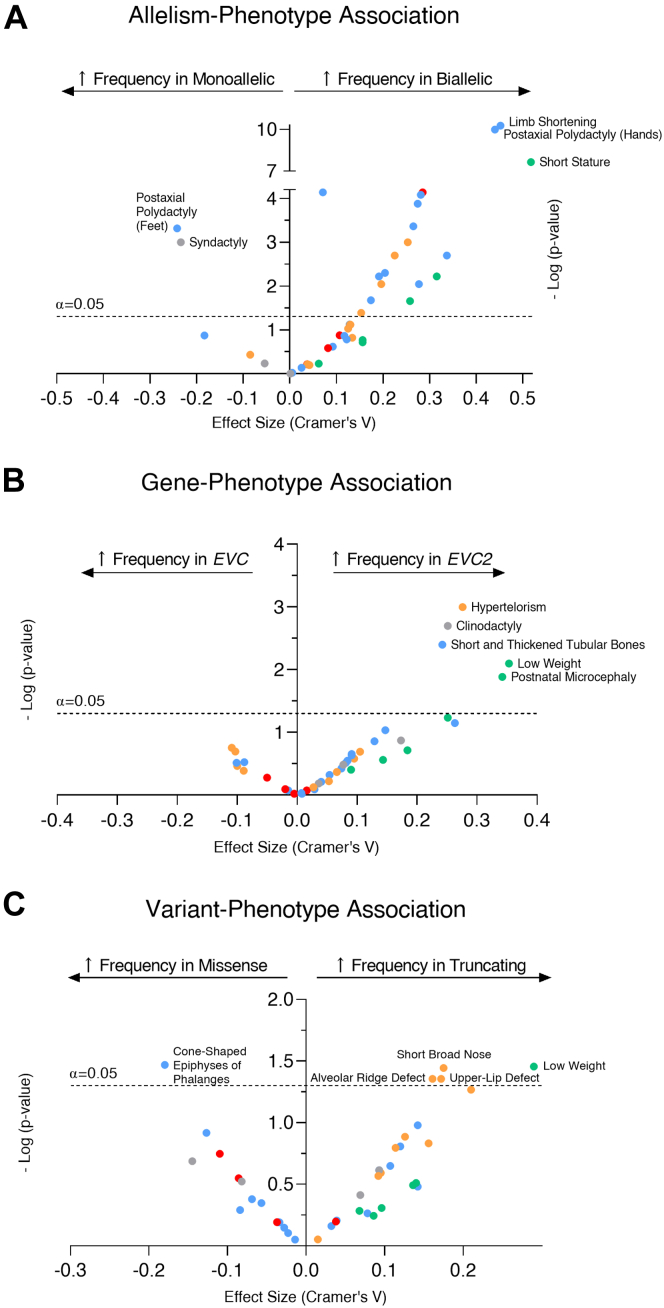
**Comparison of phenotype frequency between variant types.** (A) Plotting of the effect size of the proportion analysis vs the significance value (in a minus logarithm scale) for the association of phenotypes to allelism, (B) mutated gene, and (C) type of variant. The sign of the effect size value represents the direction of the effect. The dashed line represents the cutoff value for a 95% confidence interval. Each dot represents one specific phenotype, with colors relating to the general group in which it is included: blue for skeletal findings, red for heart conditions, green for anthropometric changes, orange for facial features, and gray for other features. *EVC*, Ellis-van Creveld.

Skeletal, cardiovascular, and thoracic phenotypes were less common in cases with monoallelic variants, but the frequency order of affected systems was exactly the same between subjects with monoallelic and biallelic variants (Table 2). The most common phenotype in subjects with monoallelic variants was, as with those with biallelic variants, postaxial polydactyly of the hands (71% of cases), which is bilateral in 90% of cases (Table 3). When we compare the proportions of each specific phenotype, we observed that a large number were significantly more frequent in biallelic cases (Figure 2A, Table 3), with short stature and limb shortening presenting the largest effects. Curiously, postaxial polydactyly of the feet was much more associated with monoallelic (60.7%) than biallelic variants (27.6%) as well as syndactyly. Although these results show that the clinical phenotype is milder in monoallelic cases, 2 specific phenotypes were underrepresented in biallelic cases.

### Characterization of prenatal cases with biallelic variants

Regarding biallelic cases with a prenatal diagnosis (*N* = 40), both genes were equally implicated, with mostly truncating variants and compound heterozygosity (Supplemental Table 1). Diagnostic tests were generally more untargeted than for postnatal cases. Regarding the clinical phenotype, at least 1 skeletal anomaly was present in all cases, the most common being postaxial polydactyly of the hands, limb shortening, or short ribs (100%, 90%, and 79%, respectively). Congenital heart defects were also common, with atrial septal defect being the most common malformation, occurring in up to 60% of cases. When compared with postnatal cases, facial features were significantly less frequent (which is expected before the limited ability to assess these features prenatally), whereas thoracic anomalies were more often detected (Supplemental Table 2). In conclusion, the clinical presentation of prenatal cases was not remarkably different than that of postnatal cases.

### Genotype-phenotype correlations of biallelic cases

Because no genotype-phenotype correlations have been established for the EVC syndrome, it is unknown whether cases associated with either *EVC* or *EVC2* have differences in the prevalence of specific phenotypes. We compared the phenotype frequencies in biallelic cases of *EVC* vs *EVC2* pathogenic variants (Figure 2B). For general phenotypes, a small effect could be observed for an increased frequency of thoracic anomalies in *EVC2* cases (Supplemental Table 3). Nevertheless, several specific phenotypes were more frequent in *EVC2* cases, mostly associated with short stature and short bone length (Supplemental Table 4). Therefore, biallelic pathogenic variants in *EVC2* possibly have a greater impact on long bone development compared with those in *EVC*.

**Figure 3:**
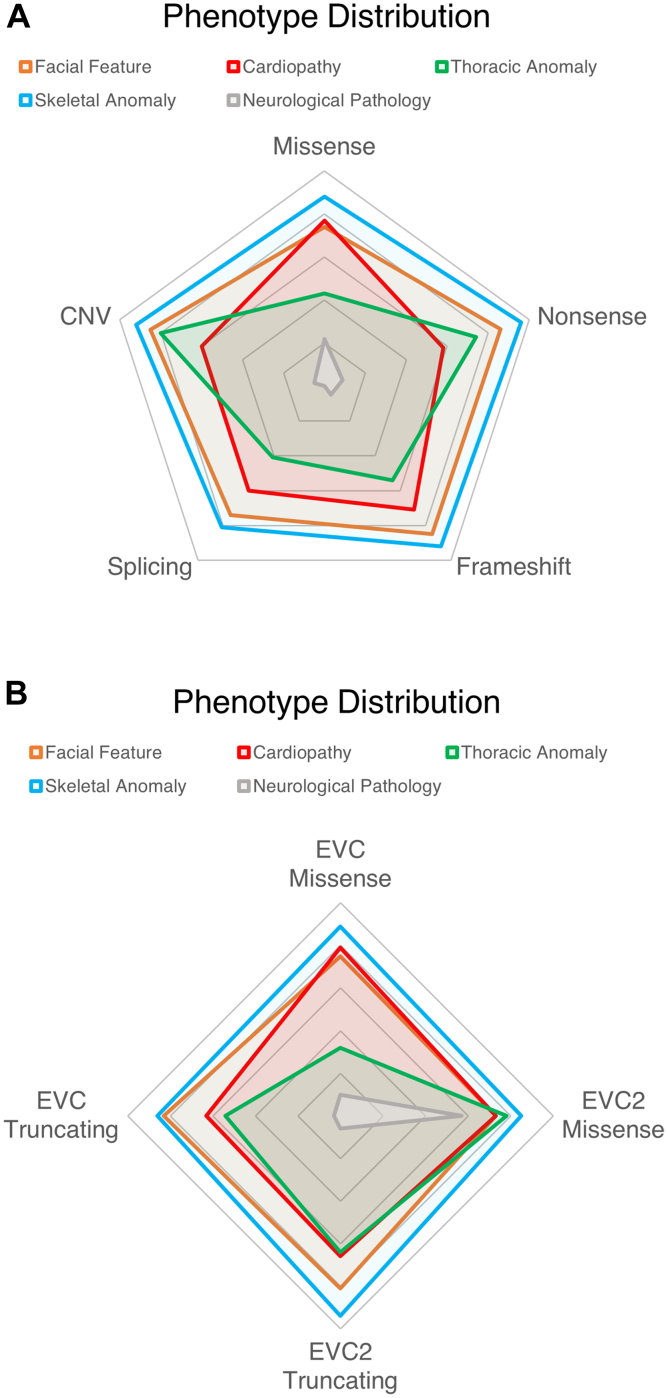
**Distribution of phenotype categories between variant types.** (A) Radial plot of the frequency of each general phenotype per subtype of variant or (B) general phenotype per gene and variant type. Each radial track represents 20% of proportion, with the outer layer corresponding to 100%. *CNV*, copy number variant; *EVC*, Ellis-van Creveld.

Then, we compared whether any general or specific phenotype was particularly more frequent in biallelic missense vs biallelic truncating (nonsense, frameshift, splicing, or CNVs) variants (Supplemental Tables 5 and 6). We excluded patients with 1 missense plus 1 truncating variant because they were very uncommon. Interestingly, patients with truncating variants had a significantly higher frequency of dysmorphic features (Figure 2C), without any other major impact in phenotypes. Nevertheless, there were no major significant differences between phenotype frequency. We further assessed variant type by comparing the phenotype frequency between patients with different types of biallelic variants (missense, nonsense, splicing, frameshift, or CNVs) (Figure 3A) and observed a significant difference for thoracic features, which are more common for nonsense variants and CNVs and less common in missense and splicing variants (Supplemental Table 7). Additionally, we evaluated for differences between specific phenotypes in different variant types to assess whether specific pathogenic variants could associate with specific disease manifestations (Supplemental Table 8). We observed that CNVs associated the most with changes in phenotype frequency and were less resemblant of the classical phenotype because postaxial polydactyly and cone-shaped phalanges were less common and dysmorphic features more frequent (Table 4). Heart conditions were the phenotype group that had the least variation among different variant types.

**Table 4:** Phenotypes with a significant increase/decrease in frequency when associated with specific types of variants

Feature Type^a^	Missense	Nonsense	Frameshift	Splicing	CNV
Skeletal		**↓** Cone-shaped phalanges		**↓** Nail dysplasia **↑** Limb shortening at birth	**↓** Postaxial polydactyly (hand) **↓** Cone-shaped phalanges
Facial	**↓** Teeth phenotypes^c^		**↑** Hypertelorism	**↓** Alveolar ridge defect	**↑** Dysmorphic features^b^ **↑** Brachydactyly
Heart				**↓** VSD	
Thorax		**↑** Narrow chest **↑** Short ribs			**↑** Narrow chest **↑** Short ribs
Other			**↑** Clinodactyly		

*CNV*, copy number variant; *VSD*, ventricular septal defect.

a Phenotypes that are significantly increased (**↑**) or decreased (**↓**) in different types of biallelic variants, based on the analysis reported in Supplemental Table 8.

b Dysmorphic features refer to upper-lip defect, short broad nose, and long philtrum.

c Teeth phenotypes refer to alveolar ridge defect and neonatal teeth.

Finally, we assessed whether phenotypes had variable frequencies when comparing both the causal gene and variant type. For this analysis, we combined truncating variants in one group because sample size was not sufficient for an adequate analysis of all subtypes of variants per gene. We observed that for general phenotypic groups, thoracic manifestations were less frequent in missense variants of the *EVC* gene when compared with truncating ones or any *EVC2* variant (Figure 3B, Supplemental Table 9). Additionally, neurologic symptoms were more frequent in *EVC2* missense variants than in any other group. No differences were observed in skeletal, heart, or facial phenotypes. When it comes to specific manifestations, it is noteworthy that *EVC* missense variants were associated with a lower incidence of phenotypes that are very common, possibly indicating a decrease severity overall (Table 5, Supplemental Table 10). As previously observed, truncating variants in either gene are associated with an increase frequency of abnormal anthropometry. Curiously, foot polydactyly was very uncommon in the *EVC2* missense group.

**Table 5:** Phenotypes with a significant increase/decrease in frequency when associated with specific types of variants/genes

Feature Type^a^	*EVC* Missense	*EVC* Truncating	*EVC2* Missense	*EVC2* Truncating
Skeletal	**↓** Short tubular bones **↓** Low iliac wings		**↓** Foot polydactyly **↑** Club foot	
Facial	**↓** Upper-lip defect			**↑** Hypertelorism
Heart				
Thorax				
Other	**↑** Syndactyly	**↑** Low weight		**↑** Low weight **↑** Postnatal microcephaly **↑** Clinodactyly

*EVC*, Ellis-van Creveld.

a Phenotypes that are significantly increased (**↑**) or decreased (**↓**) in different types of biallelic variants and gene based on the analysis reported in Supplemental Table 10.

### *CRMP1*-overlapping *EVC* variants are associated with a more severe phenotype

**Figure 4:**
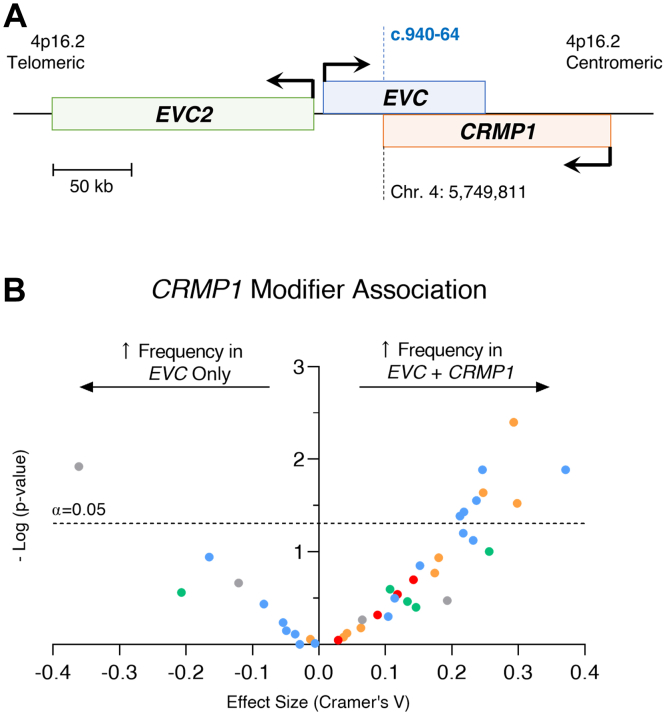
**Assessment of*****CRMP1*****as a potential modifier of EVC syndrome severity.** (A) Schematic representation of the human genome map in the area, including *EVC2*, *EVC*, and *CRMP1*. Arrows indicate transcription direction. Genomic coordinates refer to the GRCh38 version of the human genome. (B) Plotting of the effect size of the proportion analysis vs the significance value (in a minus logarithm scale) for the association of phenotypes to variants affecting *EVC* only vs *EVC* and *CRMP1*. The dashed line represents the cutoff value for a 95% confidence interval. Each dot represents one specific phenotype, with colors relating to the general group in which it is included: blue for skeletal findings, red for heart conditions, green for anthropometric changes, orange for facial features, and gray for other features. *EVC*, Ellis-van Creveld.

The distal coding region of the *EVC* gene overlaps with the distal coding region of the *CRMP1* gene, which is transcribed inversely to *EVC* (Figure 4A).^35^ It is unknown whether this 3′ overlap has any functional significance. Indeed, *CRMP1* has not been associated with any human disorder. This gene encodes for the collapsin response mediator protein-1 (CRMP1) and is expressed in developing limbs and ectoderm: combined with the shared distal protein sequence of EVC, it was suggested to have a role in processes also regulated by EVC.^36^ We compared the phenotype frequency of variants proximal to the start of the gene overlap (GRCh38, chr4: 5748084), which affect only *EVC*, and those distal to that position, which affect both *EVC* and *CRMP1*. We observed an imbalance toward variants that affected both genes, which were associated with an increased frequency of multiple phenotypes (Figure 4B), including several common skeletal and ectodermal dysplasia findings, with mostly moderate effects (Supplemental Tables 11 and 12). This suggests that *CRMP1* may act as a severity-modifier in *EVC*-associated EVC syndrome.

In addition to this assessment per individual variant, we have also compared the phenotype frequency in 3 patient groups: those with 2 variants only affecting *EVC* (*n* = 18); patients with 1 variant affecting only *EVC*, and 1 affecting both *EVC* and *CRMP1* (*n* = 16); and patients with both variants affecting both genes (*n* = 72) (Supplemental Tables 13 and 14). We observed that patients with either 1 or 2 variants affecting *CRMP1* had an increased frequency of skeletal (postaxial polydactyly of the hand, short and thickened tubular bones, and low iliac wings) and heart phenotypes when compared with cases with both variants affecting *EVC* only. It is also noteworthy that patients with both variants affecting *CRMP1* had an increase in thoracic phenotypes when compared with those with one or none. This corroborates the above findings and suggests a dose-dependent effect of the severity-modifying effects, because cases with both variants affecting *CRMP1* have more phenotypes than those with only one.

## Discussion

In this work, we performed the first systematic review of published cases of *EVC* and *EVC2*-associated EVC syndrome and have objectively and more accurately defined its clinical phenotype. Furthermore, we proposed genotype-phenotype correlations for the first time regarding the affected gene, variant type, and allelism. Finally, we have also identified *CRMP1* as a potential genetic modifier of EVC syndrome severity, providing the first clinical evidence for the implication of *CRMP1* in human disease.

We suggest that a revision of the classically described EVC syndrome features (such as in the respective OMIM entry 22550) is required because we found that genital phenotypes are very uncommon and potentially unrelated with the syndrome itself. Furthermore, we identified other features that were sufficiently common to require listing in the OMIM database, such as VSD (previously identified in multiple patients),^37^^,^^38^ additional dysmorphisms (such as short broad nose, long philtrum, and hypertelorism), and additional finger anomalies (brachydactyly, syndactyly, and clinodactyly). This objective assessment provides the best evidence for the preparation of a revised feature list of *EVC* or *EVC2*-associated cases. This is extremely important given that several clinical diagnoses of EVC syndrome are misdiagnosed because the underlying genetic cause is different and may have a different set of manifestations that require detailed characterization.

Prior literature reports have reported that monoallelic cases are less severe than biallelic ones.^23^^,^^24^ Our study clearly supports this notion, showing that monoallelic EVC (or WAD) is much less severe than the biallelic form, with a decreased frequency of all types of manifestations. This indicates that the condition is gene dosage-dependent because the presence of 1 fully functional allele is sufficient for the disease to manifest significantly more lightly. Nevertheless, penetrance for monoallelic variants is incomplete. Although we were not able to calculate penetrance with this study, we estimate it to be significantly lower than 50% because heterozygous individuals in families with biallelic cases are almost always asymptomatic.

One peculiar feature of monoallelic cases is that postaxial polydactyly of the feet is much more common when compared with patients with biallelic variants in which postaxial polydactyly of the hands is significantly more frequent. This may indicate that genetic interactions between the normal and mutated alleles, such as a dominant-negative effect, may selectively affect toe development.

Another hypothesis in prior studies is that monoallelic EVC was only associated with *EVC2* variants in exon 22.^23^ However, this review identified symptomatic cases with nonexon 22 *EVC2* variants, *EVC* variants, and one case with a CNV affecting both genes. Interestingly, more than 80% of monoallelic symptomatic cases are caused by *EVC2* pathogenic variants, which is in line with the observation that biallelic cases affecting *EVC2* are more severe than those affecting *EVC*. This increased severity is observed for different types of symptoms, such as skeletal, facial, and anthropometric, and not for a specific subset of phenotypes. In conclusion, we can infer that *EVC2* seems to be more important than *EVC* for ciliary function.

Regarding prenatal cases, most were readily identified because of anatomic anomalies in the fetal ultrasound, related with skeletal development and/or polydactyly. The differences we found in terms of phenotype frequency (compared with postnatal cases) were mild and easily explained by the obvious limitations of ultrasound assessment vs a complete ex utero physical examination. Nevertheless, it is important to observe that the EVC syndrome can often be readily identified in the prenatal setting.

Truncating variants are commonly associated with a more severe mutational effect in genes when compared with missense ones. Indeed, regardless of the affected gene, we identified that truncating variants associated with a mild-modest frequency increase in facial features and small weight. When we performed a more detailed analysis of general features by variant type and gene, we observed that skeletal and heart features (very common findings of the syndrome) were equally proportional among all groups. However, thoracic features, which are also staples of the EVC syndrome, were much less common in *EVC* missense variants. This further adds to the notion that *EVC* and missense variants are associated with milder disease severity (vs *EVC2* and/or truncating variants).

In the same line of thought, *EVC* missense cases had a decreased frequency of some specific phenotypes that are very common in the condition (namely, short tubular bones, low iliac wings, and upper lip defects). Finally, *EVC2* truncating variants were associated with an increased frequency of less common phenotypes (such as dysmorphic and anthropometric features), indicating more complex clinical presentations than those classically attributed to the EVC syndrome.

Finally, when we assessed the frequency of manifestations by variant subtype (independently of the affected gene), we observed that the proportion of the most common general features (skeletal, heart, and facial) were comparable among the 5 types of variants. Thoracic features, which seem to vary more according to variant type (as stated above), are also increased in nonsense and CNVs, which may reflect a more deleterious effect for the respective proteins. However, other variant types were also associated with slightly different presentations. Nevertheless, it would be important to assess variant subtype per gene to further dissect these findings, hopefully in a future review that includes more patients to allow for such analysis.

The significant overlap between the coding sequence of *EVC* and *CRMP1* indicates that they act in similar cellular processes. However, *CRMP1* has never been associated with human disease. Our phenotypic association findings indicate that *EVC* pathogenic variants that also affect the common coding region with *CRMP1* lead to increased severity of the clinical findings in a dose-dependent manner. Interestingly, knockout of the mouse ortholog *Crmp1* leads to impairment in neuronal migration, proliferation and spine density, culminating in impaired spatial memory and electrophysiological stimuli transmission.^39^, ^40^, ^41^ This gene has, in fact, been proposed as a potential therapeutical target for Alzheimer disease and amyotrophic lateral sclerosis.^42^^,^^43^ In addition, CRMP1 has also been implicated in tumor invasion suppression.^44^ All these findings relate with the ubiquitous function of CRMP1, which acts as an intracellular mediator for semaphorin 3A signaling, largely through binding of CRMP1 to F-actin and microtubules.^44^ Therefore, EVC-dependent signaling in the microtubules of cilia may be further compromised if CRMP1 signaling, which is also dependent on microtubule binding, is also defective. This interaction is well established in neurons, but further evidence is required for its implication in ectodermal development because the *CRMP1* gene is expressed in multiple peripheral tissues. Interestingly, this gene has been implicated in odontoblast morphogenesis.^45^

One of the main limitations of this work is the bias associated with phenotype reporting in the published cases. Because the condition was more and more understandable, perhaps clinicians would be more sensible to other findings and therefore report them. Nevertheless, most cases we included in the analysis were relatively recent because they had to include a molecular diagnosis, which was only possible in the 21st century. Furthermore, another limitation is that the conclusions from the analytical work are correlational and not causal and require validation in the future. However, these studies make the best of the available literature and are indispensable to generate data-based hypothesis.

In summary, we established that (1) monoallelic *EVC*/*EVC2* variants have incomplete penetrance and less phenotypic expressivity than biallelic ones; (2) *EVC* variants associate with a less severe clinical presentation than *EVC2* variants, especially if they are nontruncating; (3) thoracic anomalies are usually more variable with the implicated gene/variant types; and (4) *CRMP1* is the first identified potential genetic modifier of severity in *EVC-*associated EVC syndrome. We hope to finally provide a clear picture of the molecular EVC syndrome, which should be differentiated from other EVC-like conditions with a different genetic cause, which are ultimately different entities.

## Data Availability

All produced data are available in the manuscript or supplementary material. The protocol for the review, which follows COSMO-E guidelines where applicable for case reports/series, can be accessed in the supplementary material. There was no formal registration of this systematic review because it was directed only to case reports/series. The file with all extracted data is available in supplementary material.
