# Supplementary material for: Establishing an objective clinical spectrum, genotype-phenotype correlations, and CRMP1 as a modifier in the Ellis-van Creveld syndrome: The first systematic review of EVC- and EVC2-associated conditions
Source: Genet Med Open. 2023 Mar 13;1(1):100781. doi: 10.1016/j.gimo.2023.100781 (PMC11613718; doi:10.1016/j.gimo.2023.100781)
Supplement: Supplementary Table [file mmc1.pdf]

Supplementary Tables

Supplementary Table 1. Characterization of prenatal subjects with biallelic variants.

Variable		**Prenatal N (%)**[a]	
**Sex**	Male	23	(62.2%)
	Female	14	(37.8%)
**Ethnicity**	African	2	(5.4%)
	Arab	4	(10.8%)
	Asian	6	(16.2%)
	White	25	(67.6%)
**Positive Family History**		2	(11.1%)
**Consanguinity**		8	(20.5%)
**Affected Gene**	*EVC*	19	(47.5%)
	EVC2	19	(47.5%)
	Both[b]	2	(5.0%)
**Allelism**	Homozygous	11	(27.5%)
	Compound Heterozygous	29	(72.5%)
**Variant Type**	Missense	5	(6.3%)
	Nonsense	20	(25.0%)
	Frameshift	22	(27.5%)
	Splicing	14	(17.5%)
	CNV	19	(23.8%)
**Variant Combination**	Missense Missense	1	(2.5%)
	Missense Truncating	3	(7.5%)
	Truncating Truncating	36	(90.0%)
**Diagnostic Test**	Sanger (Targeted)	14	(35.0%)
	NGS Panel	13	(32.5%)
	WES/WGS	4	(10.0%)
	Array	2	(5.0%)
	MLPA	2	(5.0%)
	Multiple	5	(12.5%)

NA, not applicable.
[a] Phenotype frequency is represented by the absolute number (N) and respective proportion (%).
[b] Cases with “both” affected genes have either 1 or 2 copy number variants that affect both *EVC* and *EVC2* in cis, as there is no case with digenic inheritance.

Supplementary Table 2. Clinical phenotype of prenatal subjects with biallelic variants.

Phenotype by System	%	(N/Total)[a]
Skeletal Anomaly	100%	(40/40)
Skeletal Anomaly (excluding polydactyly)	93%	(37/40)
Thoracic Anomaly	80%	(31/39)
Cardiopathy	73%	(29/40)
Facial Feature	50%	(18/36)
Genital Anomaly	4%	(1/23)

Specific Phenotypes	%	(N/Total)[a]
Postaxial Polydactyly (Hand)	100%	(40/40)
Bilateral	100%	(40/40)
Limb Shortening	90%	(36/40)
Short Ribs	79%	(31/39)
Narrow Chest	77%	(30/39)
Other Phenotypes	60%	(15/25)
Short and Thickened Tubular Bones	63%	(25/40)
Other Dysmorphisms	60%	(15/25)
Atrial Septal Defect	60%	(24/40)
Alveolar Ridge Defect	50%	(8/16)
Other Congenital Heart Defects	50%	(19/38)
Low Iliac Wings	50%	(20/40)
Acetabula Spur Projections	48%	(19/40)
Nail Dysplasia/Hypoplasia	47%	(14/30)
Short Broad Nose	44%	(11/25)
Brachydactyly	35%	(14/40)
Ventricular Septal Defect	32%	(12/38)
Long Philtrum	28%	(7/25)
Upper-Lip Defect	26%	(9/34)
Postaxial Polydactyly (Foot)	15%	(6/40)
Bilateral	100%	(6/6)
Club Foot	13%	(5/40)
Cleft Lip	12%	(4/33)
Syndactyly	10%	(4/40)
Lung Hypoplasia	10%	(4/40)

[a] Phenotype frequency is represented by the absolute number (N) for the patients in which the phenotype was assessed (Total), with the respective proportion (%).

Supplementary Table 3. General phenotypes (by system) in patients with *EVC* or *EVC2* biallelic variants.

Feature	***EVC*** **% (N/Total)**[a]	***EVC2*** **% (N/Total)**[a]	Chi-square	Sig. (p)[b]	Cramer's V
Skeletal Anomaly	99.0% (103/104)	100.0% (77/77)	0.744	0.388	0.064
Skeletal Anomaly (excluding polydactyly)	86.3% (88/102)	93.4% (71/76)	2.333	0.127	0.114
Facial Feature	82.0% (73/89)	80.0% (60/75)	0.109	0.742	0.026
Congenital Heart Disease	65.2% (58/89)	67.1% (49/73)	0.068	0.794	0.021
Thoracic Anomaly	50.5% (47/93)	65.2% (45/69)	3.479	0.062	0.147
Neurological Disease	4.1% (3/74)	9.4% (5/53)	1.514	0.218	0.109
Genital Anomaly	8.7% (4/46)	3.1% (1/32)	0.976	0.323	0.112

[a] Phenotype frequency is represented by the absolute number (N) for the patients in which the phenotype was assessed (Total), with the respective proportion (%).
[b] Significance values in bold mean that $p < 0.050$.

Supplementary Table 4. Specific phenotypes in patients with *EVC* or *EVC2* biallelic variants.

Feature	***EVC*** **% (N/Total)**[a]	***EVC2*** **% (N/Total)**[a]	Chi-square	Sig. (p)[b]	Cramer's V
Postaxial Polydactyly (Hands)	98.1% (102/104)	100.0% (77/77)	1.497	0.221	0.091
Bilateral	99.0% (101/102)	98.7% (76/77)	0.040	0.841	0.015
Short Stature	74.5% (38/51)	82.1% (32/39)	0.727	0.394	0.090
Limb Shortening	75.8% (75/99)	80.3% (61/76)	0.504	0.478	0.054
Nail Dysplasia/Hypoplasia	68.6% (59/86)	75.4% (43/57)	0.783	0.376	0.074
Hypodontia	63.2% (36/52)	68.3% (28/41)	0.278	0.598	0.053
Alveolar Ridge Defect	54.7% (41/75)	64.1% (41/64)	1.260	0.262	0.095
Atrial Septal Defect	56.2% (50/89)	55.7% (39/70)	0.003	0.953	0.005
Narrow Chest	49.5% (46/93)	58.0% (40/69)	1.151	0.283	0.084
Short Ribs	48.4% (44/91)	56.5% (39/69)	1.049	0.306	0.081
Short and Thickened Tubular Bones	40.0% (30/75)	64.2% (43/67)	8.282	**0.004**	0.242
Delayed Eruption of Teeth	51.0% (25/49)	42.1% (16/38)	0.683	0.409	0.089
Other Dysmorphisms	31.6% (25/79)	41.8% (28/67)	1.614	0.204	0.105
Other Congenital Heart Defects	39.1% (34/87)	34.2% (25/73)	0.398	0.528	0.050
Limb Shortening at Birth	35.6% (16/45)	36.4% (8/22)	0.004	0.948	0.008
Low Iliac Wings	26.1% (18/69)	38.1% (24/63)	2.189	0.139	0.129
Low Weight	18.4% (7/38)	52.6% (10/19)	7.083	**0.008**	0.353
Acetabula Spur Projections	21.7% (15/54)	34.9% (22/63)	2.836	0.092	0.147
Postaxial Polydactyly (Feet)	23.5% (23/98)	31.6% (24/76)	1.428	0.232	0.091
Bilateral	78.3% (18/23)	95.8% (23/24)	3.257	0.071	0.263
Pre-Natal Limb Shortening	25.0% (10/40)	27.6% (8/29)	0.058	0.809	0.029

Upper-Lip Defect	28.6% (24/84)	19.7% (14/71)	1.630	0.202	0.103
Genu Valgum	20.0% (17/85)	23.3% (17/73)	0.251	0.616	0.040
Abnormal Birth Stature	17.1% (6/35)	29.2% (7/24)	1.198	0.274	0.143
Brachydactyly	19.3% (17/88)	22.2% (16/72)	0.204	0.652	0.036
Neonatal Teeth	24.5% (13/53)	16.2% (6/37)	0.904	0.342	0.100
Short Broad Nose	17.1% (13/76)	22.4% (15/67)	0.631	0.427	0.066
Ventricular Septal Defect	19.3% (16/83)	20.6% (14/68)	0.040	0.841	0.016
Cone-Shaped Epiphyses of Phalanges	20.0% (15/75)	18.5% (12/65)	0.053	0.818	0.019
Syndactyly	12.5% (11/88)	18.1% (13/72)	0.959	0.328	0.077
Capitate-Hamate Fusion	20.3% (15/74)	13.6% (9/66)	1.081	0.299	0.088
Long Philtrum	14.5% (11/76)	16.4% (11/67)	0.103	0.748	0.027
Cleft Lip	14.1% (12/85)	7.2% (5/69)	1.831	0.176	0.109
Single Atrium	11.2% (10/89)	10.0% (7/70)	0.063	0.802	0.020
Low Birth Weight	2.9% (1/34)	17.4% (4/23)	3.580	0.058	0.251
Postnatal Microcephaly	0.0% (0/35)	16.7% (3/18)	6.183	**0.013**	0.342
Developmental Delay	5.9% (3/51)	16.7% (4/24)	2.243	0.134	0.173
Prenatal Microcephaly	3.6% (1/28)	13.6% (3/22)	1.696	0.193	0.184
Pectus Carinatum	10.2% (6/59)	4.7% (2/43)	1.048	0.306	0.101
Clinodactyly	1.1% (1/88)	13.9% (10/73)	10.059	**0.002**	0.251
Hypertelorism	0.0% (0/76)	13.4% (9/67)	10.895	**0.001**	0.276
Club Foot	4.5% (4/88)	4.8% (3/62)	0.007	0.933	0.007

[a] Phenotype frequency is represented by the absolute number (N) for the patients in which the phenotype was assessed (Total), with the respective proportion (%).
[b] Significance values in bold mean that $p < 0.050$.

Supplementary Table 5. General phenotypes (by system) in patients with missense or truncating biallelic variants.

Feature	**Missense %(N/Total)[a]**	**Truncating %(N/Total)[a]**	Chi-square	Sig. (p)[b]	Cramer's V
Skeletal Anomaly	100.0% (17/17)	99.4% (161/162)	0.106	0.745	0.024
Skeletal Anomaly (excluding polydactyly)	87.5% (14/16)	90.0% (144/160)	0.099	0.753	0.024
Facial Feature	76.5% (13/17)	82.8% (120/145)	0.409	0.522	0.050
Congenital Heart Disease	70.6% (12/17)	61.5% (88/143)	0.531	0.466	0.058
Thoracic Anomaly	50.0% (7/14)	61.0% (89/146)	0.639	0.424	0.063
Neurological Disease	27.3% (3/11)	4.3% (5/117)	9.077	**0.003**	0.266
Genital Anomaly	11.1% (1/9)	5.8% (4/69)	0.375	0.540	0.069

[a] Phenotype frequency is represented by the absolute number (N) for the patients in which the phenotype was assessed (Total), with the respective proportion (%).
[b] Significance values in bold mean that $p < 0.050$.

Supplementary Table 6. Specific phenotypes in patients with missense or truncating biallelic variants.

Feature	**Missense %(N/Total)**	**Truncating %(N/Total)**	Chi-square	Sig. (p)	Cramer's V
Postaxial Polydactyly (Hand)	100.0% (17/17)	98.8% (160/162)	0.212	0.645	0.034
Bilateral	100.0% (17/17)	98.8% (158/160)	0.215	0.643	0.035
Short Stature	72.7% (8/11)	81.0% (64/79)	0.414	0.520	0.068
Limb Shortening	87.5% (14/16)	79.6% (125/157)	0.571	0.450	0.057
Nail Dysplasia/Hypoplasia	76.5% (13/17)	73.4% (91/124)	0.073	0.786	0.023
Hypodontia	64.3% (9/14)	66.3% (55/83)	0.021	0.885	0.015
Alveolar Ridge Defect	35.7% (5/14)	63.4% (78/123)	4.039	**0.044**	0.172
Atrial Septal Defect	66.7% (10/15)	52.1% (74/142)	1.155	0.282	0.086
Narrow Chest	50.0% (7/14)	56.8% (83/146)	0.244	0.622	0.039
Short Ribs	50.0% (7/14)	55.6% (80/144)	0.159	0.690	0.032
Short and Thickened Tubular Bones	33.3% (4/12)	54.7% (70/128)	1.242	0.156	0.120
Delayed Eruption of Teeth	27.3% (3/11)	50.7% (38/75)	2.105	0.147	0.156
Other Dysmorphisms	18.8% (3/16)	38.0% (49/129)	2.290	0.130	0.126
Other Congenital Heart Defects	41.2% (7/17)	35.5% (50/141)	0.215	0.643	0.037
Limb Shortening at Birth	25.0% (1/4)	40.4% (23/57)	0.369	0.544	0.078
Low Iliac Wings	16.7% (2/12)	33.9% (40/118)	1.479	0.224	0.107
Low Weight	0.0% (0/8)	37.8% (17/45)	4.449	**0.035**	0.290
Acetabula Spur Projections	8.3% (1/12)	30.5% (36/118)	2.631	0.105	0.142
Postaxial Polydactyly (Foot)	31.3% (5/16)	26.9% (42/156)	0.137	0.711	0.028
Bilateral	80.0% (4/5)	92.9% (39/42)	0.949	0.330	0.142
Pre-Natal Limb Shortening	40.0% (2/5)	26.3% (15/57)	0.433	0.511	0.084
Upper-Lip Defect	6.3% (1/16)	30.0% (42/140)	4.057	**0.044**	0.161
Genu Valgum	12.5% (2/16)	25.7% (36/140)	1.361	0.243	0.093
Abnormal Birth Stature	0.0% (0/3)	26.0% (13/50)	1.034	0.309	0.140
Brachydactyly	13.3% (2/15)	23.1% (33/143)	0.747	0.387	0.069
Neonatal Teeth	0.0% (0/11)	26.0% (19/73)	3.700	0.054	0.210
Short Broad Nose	0.0% (0/16)	22.0% (28/127)	4.386	**0.036**	0.175

Ventricular Septal Defect	33.3% (5/15)	18.7% (25/134)	1.807	0.179	0.110
Cone-Shaped Epiphyses of Phalanges	41.7% (5/12)	16.7% (21/126)	4.478	**0.034**	0.180
Syndactyly	26.7% (4/15)	16.1% (23/143)	1.073	0.300	0.082
Capitate-Hamate Fusion	25.0% (3/12)	15.9% (20/126)	0.657	0.418	0.069
Long Philtrum	6.3% (1/16)	17.3% (22/127)	1.291	0.256	0.095
Cleft Lip	0.0% (0/15)	11.8% (16/136)	1.974	0.160	0.114
Single Atrium	6.7% (1/15)	10.6% (15/142)	0.225	0.635	0.038
Low Birth Weight	0.0% (0/4)	10.6% (5/47)	0.472	0.492	0.096
Postnatal Microcephaly	0.0% (0/8)	11.1% (5/45)	0.981	0.322	0.136
Developmental Delay	20.0% (2/10)	7.6% (5/66)	1.603	0.205	0.145
Prenatal Microcephaly	0.0% (0/3)	9.8% (4/41)	0.322	0.570	0.086
Pectus Carinatum	9.1% (1/11)	7.9% (7/89)	0.020	0.888	0.014
Clinodactyly	0.0% (0/15)	8.4% (12/143)	1.362	0.243	0.093
Hypertelorism	0.0% (0/16)	7.1% (9/127)	1.210	0.271	0.092
Club Foot	12.5% (2/16)	3.8% (5/132)	2.404	0.121	0.127

[a] Phenotype frequency is represented by the absolute number (N) for the patients in which the phenotype was assessed (Total), with the respective proportion (%).

[b] Significance values in bold mean that $p < 0.050$.

Supplementary Table 7. General phenotypes (by system) in patients with different types of genetic variants.

Feature[a]	**Missense (N/Total)**	**Nonsense (N/Total)**	**Frameshift (N/Total)**	**Splicing (N/Total)**	**CNV (N/Total)**	Adjusted Chi-square[b]	Adjusted Sig. (p)[c]	Adjusted Cramer's V
Skeletal Anomaly	100% (43/43)	100% (84/84)	100% (91/91)	100% (106/106)	96.2% (50/52)	6.303	0.177	0.183
Skeletal Anomaly (excluding polydactyly)	87.8% (36/41)	96.4% (81/84)	92.3% (84/91)	81.4% (83/102)	92.3% (48/52)	6.053	0.195	0.188
Facial Feature	74.4% (32/43)	86.3% (69/80)	85.2% (75/88)	74.1% (63/85)	84.8% (39/46)	3.055	0.549	0.141
Congenital Heart Disease	76.7% (33/43)	57.9% (44/76)	70.8% (63/89)	60.3% (47/78)	59.6% (31/52)	3.830	0.430	0.143
Thoracic Anomaly	43.2% (16/37)	74.1% (60/81)	53.6% (45/84)	40.7% (35/86)	80.0% (40/50)	16.390	**0.003**	0.313

[a] Phenotype frequency is represented by the absolute number (N) for the patients in which the phenotype was assessed (Total), with the respective proportion (%).
[b] An adjusted Chi-square test was used to account for the virtual duplication of sample size. Note that neurological and genital phenotypes were not included as there were no cases in at least one of the categories of variants.
[c] Significance values in bold mean that $p < 0.050$.

Supplementary Table 8. Specific phenotypes in patients with different types of genetic variants.

Feature[a]	**Missense (N/Total)**	**Nonsense (N/Total)**	**Frameshift (N/Total)**	**Splicing (N/Total)**	**CNV (N/Total)**	Adjusted Chi-square[b]	Adjusted Sig. (p)[c]	Adjusted Cramer's V
Postaxial Polydactyly (Hand)	100% (43/43)	100% (84/84)	100% (91/91)	100% (106/106)	92.3% (48/52)	12.670	**0.013**	0.259
Bilateral	100% (43/43)	100% (84/84)	100% (91/91)	98.1% (104/106)	95.8% (46/48)	3.700	0.448	0.140
Short Stature	69.2% (18/26)	91.1% (41/45)	69.1% (38/55)	82.5% (33/40)	81.8% (18/22)	4.853	0.303	0.219
Limb Shortening	78.0% (32/41)	88.1% (74/84)	71.4% (65/91)	74.0% (71/96)	84.6% (44/52)	4.869	0.301	0.163
Nail Dysplasia/Hypoplasia	69.8% (30/43)	84.2% (64/76)	76.3% (45/59)	50.0% (37/74)	83.3% (40/48)	13.850	**0.008**	0.300
Hypodontia	64.7% (22/34)	79.2% (42/53)	66.7% (36/54)	52.3% (23/44)	61.9% (13/21)	3.655	0.455	0.197
Alveolar Ridge Defect	40.0% (14/35)	70.0% (49/70)	72.7% (56/77)	40.6% (28/69)	73.0% (27/37)	14.240	**0.007**	0.309
Atrial Septal Defect	69.2% (27/39)	51.3% (39/76)	52.9% (46/87)	55.1% (43/78)	51.9% (27/52)	2.268	0.687	0.109
Narrow Chest	43.2% (16/37)	67.9% (55/81)	48.8% (41/84)	39.5% (34/86)	76.0% (38/50)	13.420	**0.009**	0.277
Short Ribs	43.2% (16/37)	72.2% (57/79)	40.5% (34/84)	36.9% (31/84)	80.0% (40/50)	21.290	**<0.001**	0.353
Short and Thickened Tubular Bones	27.3% (9/33)	45.1% (37/82)	60.8% (48/79)	42.6% (48/79)	66.0% (33/50)	7.890	0.096	0.243
Delayed Eruption of Teeth	35.7% (10/28)	51.3% (20/39)	48.1% (26/54)	50.0% (21/42)	61.9% (13/21)	1.956	0.744	0.138
Other Dysmorphisms	22.5% (9/40)	37.5% (27/72)	43.8% (35/80)	19.7% (14/71)	58.1% (25/43)	11.840	**0.019**	0.272
Other Congenital Heart Defects	39.5% (17/43)	37.3% (28/75)	37.5% (33/88)	24.4% (19/78)	46.0% (23/50)	3.552	0.470	0.146
Limb Shortening at Birth	14.3% (2/14)	33.3% (9/27)	20.7% (6/29)	54.9% (28/51)	23.1% (3/13)	14.780	**0.005**	0.332
Low Iliac Wings	18.2% (6/33)	37.7% (29/77)	18.7% (14/75)	36.4% (16/44)	46.9% (23/49)	8.723	0.068	0.237
Low Weight	0.0% (0/20)	33.3% (12/36)	42.3% (11/26)	31.6% (6/19)	38.5% (5/13)	5.970	0.201	0.313
Acetabula Spur Projections	12.1% (4/33)	33.8% (26/77)	18.7% (14/75)	25.0% (11/44)	46.9% (23/49)	9.468	0.050	0.251
Postaxial Polydactyly (Foot)	31.7% (13/41)	28.6% (24/84)	39.6% (36/91)	17.0% (16/94)	21.2% (11/52)	6.404	0.171	0.191
Bilateral	69.2% (9/13)	87.5% (21/24)	97.2% (35/36)	75.0% (12/16)	100% (11/11)	4.425	0.351	0.336
Pre-Natal Limb Shortening	33.3% (6/18)	28.9% (11/38)	16.7% (6/36)	24.1% (7/29)	42.1% (8/19)	2.049	0.727	0.183

Upper-Lip Defect	5.3% (2/38)	25.7% (19/74)	12.6% (11/87)	38.0% (30/79)	52.2% (24/46)	18.250	**0.001**	0.344
Genu Valgum	9.8% (4/41)	29.3% (24/82)	33.7% (29/86)	14.3% (10/70)	17.6% (9/51)	7.734	0.102	0.215
Abnormal Birth Stature	0.0% (0/12)	18.5% (5/27)	21.2% (7/33)	34.3% (12/35)	18.2% (2/11)	3.040	0.551	0.239
Brachydactyly	12.8% (5/39)	18.3% (15/82)	19.3% (16/83)	15.4% (12/78)	46.2% (24/52)	10.870	**0.028**	0.262
Neonatal Teeth	0.0% (0/28)	20.5% (8/39)	9.3% (4/43)	38.2% (21/55)	33.3% (5/15)	12.230	**0.016**	0.350
Short Broad Nose	2.6% (1/38)	28.6% (20/70)	17.7% (14/79)	7.2% (5/69)	41.9% (18/43)	13.570	**0.009**	0.324
Ventricular Septal Defect	28.2% (11/39)	22.7% (17/75)	17.1% (14/82)	4.3% (3/70)	34.0% (17/50)	9.752	**0.045**	0.249
Cone-Shaped Epiphyses of Phalanges	33.3% (11/33)	8.8% (7/80)	26.3% (20/76)	25.0% (14/56)	4.1% (2/49)	10.930	**0.027**	0.270
Syndactyly	20.5% (8/39)	13.4% (11/82)	24.1% (20/83)	6.4% (5/78)	19.2% (10/52)	4.455	0.348	0.179
Capitate-Hamate Fusion	21.2% (7/33)	19.8% (16/81)	18.2% (14/77)	20.4% (11/54)	0.0% (0/49)	6.319	0.177	0.199
Long Philtrum	5.1% (2/39)	24.3% (17/70)	7.6% (6/79)	5.8% (4/69)	39.5% (17/43)	19.330	**<0.001**	0.343
Cleft Lip	2.6% (1/39)	9.5% (7/74)	4.7% (4/85)	17.7% (14/79)	18.6% (8/43)	5.381	0.250	0.201
Single Atrium	7.7% (3/39)	11.8% (9/76)	9.2% (8/87)	15.4% (12/78)	3.8% (2/52)	2.484	0.648	0.125
Low Birth Weight	0.0% (0/14)	7.7% (2/26)	6.3% (2/32)	13.8% (4/29)	15.4% (2/13)	5.084	0.279	0.169
Postnatal Microcephaly	0.0% (0/20)	0.0% (0/37)	17.4% (4/23)	12.5% (2/16)	22.2% (4/18)	5.988	0.200	0.324
Developmental Delay	16.7% (4/24)	13.5% (5/37)	11.1% (3/27)	0.0% (0/50)	9.1% (2/22)	4.421	0.352	0.223
Prenatal Microcephaly	0.0% (0/12)	11.5% (3/26)	10.0% (3/30)	9.5% (2/21)	0.0% (0/11)	1.813	0.770	0.163
Pectus Carinatum	7.1% (2/28)	12.2% (6/49)	3.3% (2/61)	11.1% (6/54)	0.0% (0/20)	2.875	0.580	0.165
Clinodactyly	0.0% (0/39)	1.2% (1/82)	24.1% (20/83)	1.3% (1/78)	3.8% (2/52)	20.870	**<0.001**	0.379
Hypertelorism	0.0% (0/39)	0.0% (0/70)	20.3% (16/79)	0.0% (0/69)	4.7% (2/43)	19.970	**<0.001**	0.365
Club Foot	12.2% (5/41)	2.4% (2/83)	3.2% (2/63)	5.3% (4/76)	5.9% (3/51)	4.369	0.358	0.139

[a] Phenotype frequency is represented by the absolute number (N) for the patients in which the phenotype was assessed (Total), with the respective proportion (%).
[b] An adjusted Chi-square test was used to account for the virtual duplication of sample size.
[c] Significance values in bold mean that $p < 0.050$.

Supplementary Table 9. General phenotypes (by system) in patients with different types of genetic variants and causal gene.

Feature[a]	***EVC* Missense (N/Total)**	***EVC* Truncating (N/Total)**	***EVC2* Missense (N/Total)**	***EVC2* Truncating (N/Total)**	Adjusted Chi-square[b]	Adjusted Sig. (p)[c]	Adjusted Cramer's V
Skeletal Anomaly	100% (28/28)	98.9% (178/180)	100% (15/15)	100% (138/138)	1.017	0.797	0.075
Skeletal Anomaly (excluding polydactyly)	89.3% (25/28)	85.8% (151/176)	84.6% (11/13)	94.2% (130/138)	3.220	0.359	0.130
Facial Feature	75.0% (21/28)	83.3% (125/150)	73.3% (11/15)	80.6% (108/134)	0.923	0.819	0.074
Congenital Heart Disease	78.6% (22/28)	62.7% (94/150)	73.3% (11/15)	66.2% (86/130)	1.648	0.649	0.098
Thoracic Anomaly	32.1% (9/28)	53.8% (85/158)	77.8% (7/9)	64.1% (82/128)	7.199	0.066	0.192
Neurological Disease	10.0% (2/20)	3.1% (4/128)	57.1% (4/7)	6.1% (6/98)	14.270	**0.003**	0.362

[a] Phenotype frequency is represented by the absolute number (N) for the patients in which the phenotype was assessed (Total), with the respective proportion (%).
[b] An adjusted Chi-square test was used to account for the virtual duplication of sample size. Note that genital phenotypes were not included as there were no cases in at least one of the categories of variants.
[c] Significance values in bold mean that $p < 0.050$.

Supplementary Table 10. Specific phenotypes in patients with different types of genetic variants.

Feature[a]	***EVC* Missense (N/Total)**	***EVC* Truncating (N/Total)**	***EVC2* Missense (N/Total)**	***EVC2* Truncating (N/Total)**	Adjusted Chi-square[b]	Adjusted Sig. (p)[c]	Adjusted Cramer's V
Postaxial Polydactyly (Hand)	100% (28/28)	97.8% (176/180)	100% (15/15)	100% (138/138)	2.045	0.563	0.106
Bilateral	100% (28/28)	98.9% (174/176)	100% (15/15)	98.6% (136/138)	0.318	0.957	0.042
Short Stature	63.6% (14/22)	77.5% (62/80)	100% (4/4)	80.8% (59/73)	2.080	0.556	0.151
Limb Shortening	75.0% (21/28)	75.9% (129/170)	84.6% (11/13)	79.7% (110/138)	0.792	0.852	0.057
Nail Dysplasia/Hypoplasia	82.1% (23/28)	66.0% (95/144)	46.7% (7/15)	79.6% (78/98)	5.040	0.169	0.199
Hypodontia	69.2% (18/26)	61.4% (54/88)	50.0% (4/8)	70.3% (52/74)	1.206	0.752	0.111
Alveolar Ridge Defect	50.0% (13/26)	55.6% (69/124)	11.1% (1/9)	68.6% (81/118)	6.088	0.107	0.229
Atrial Septal Defect	71.4% (20/28)	53.3% (80/150)	63.6% (7/11)	54.7% (70/128)	1.890	0.595	0.105
Narrow Chest	32.1% (9/28)	52.5% (83/158)	77.8% (7/9)	56.3% (72/128)	4.072	0.254	0.154
Short Ribs	32.1% (9/28)	51.3% (79/154)	77.8% (7/9)	54.7% (70/128)	3.864	0.277	0.150
Short and Thickened Tubular Bones	16.7% (4/24)	44.4% (56/126)	55.6% (5/9)	65.3% (81/124)	11.880	**0.008**	0.289
Delayed Eruption of Teeth	36.4% (8/22)	55.3% (42/76)	33.3% (2/6)	42.9% (30/70)	2.006	0.571	0.152
Other Dysmorphisms	14.8% (4/27)	35.1% (46/131)	38.5% (5/13)	42.5% (51/120)	4.168	0.244	0.160
Other Congenital Heart Defects	35.7% (10/28)	39.7% (58/146)	46.7% (7/15)	32.3% (42/130)	1.438	0.697	0.085
Limb Shortening at Birth	16.7% (2/12)	38.5% (30/78)	0.0% (0/2)	36.6% (15/41)	1.681	0.641	0.157
Low Iliac Wings	4.2% (1/23)	30.7% (35/114)	55.6% (5/9)	37.1% (43/116)	8.219	**0.042**	0.216
Low Weight	0.0% (0/18)	24.1% (14/58)	0.0% (0/2)	54.3% (19/35)	10.390	**0.016**	0.417
Acetabula Spur Projections	4.2% (1/24)	25.4% (29/114)	33.3% (3/9)	35.3% (41/116)	4.693	0.196	0.198
Postaxial Polydactyly (Foot)	46.4% (13/28)	19.6% (33/168)	0.0% (0/13)	34.8% (48/138)	9.698	**0.021**	0.234
Bilateral	69.2% (9/13)	81.8% (27/33)	NA	95.8% (46/48)	NA	NA	NA
Pre-Natal Limb Shortening	28.6% (4/14)	24.2% (16/66)	50.0% (2/4)	26.4% (14/53)	0.674	0.879	0.099

Upper-Lip Defect	0.0% (0/26)	33.8% (48/142)	16.7% (2/12)	20.2% (26/129)	8.447	**0.038**	0.233
Genu Valgum	14.3% (4/28)	21.1% (30/142)	0.0% (0/13)	25.8% (34/132)	3.062	0.382	0.136
Abnormal Birth Stature	0.0% (0/10)	20.0% (12/60)	0.0% (0/2)	31.1% (14/45)	2.713	0.438	0.220
Brachydactyly	4.2% (1/24)	21.7% (33/152)	26.7% (4/15)	21.9% (28/128)	1.549	0.671	0.119
Neonatal Teeth	0.0% (0/20)	30.2% (26/86)	0.0% (0/8)	16.9% (11/65)	6.065	0.109	0.266
Short Broad Nose	0.0% (0/26)	20.6% (26/126)	7.7% (1/13)	24.2% (29/120)	4.189	0.242	0.179
Ventricular Septal Defect	28.6% (8/28)	17.4% (24/138)	27.3% (3/11)	20.2% (25/124)	1.581	0.664	0.086
Cone-Shaped Epiphyses of Phalanges	45.8% (11/24)	15.1% (19/126)	0.0% (0/9)	20.0% (24/120)	7.647	0.054	0.228
Syndactyly	33.3% (8/24)	9.2% (14/152)	0.0% (0/15)	20.3% (26/128)	7.990	**0.046**	0.222
Capitate-Hamate Fusion	29.2% (7/24)	18.5% (23/124)	0.0% (0/9)	14.8% (18/122)	3.054	0.383	0.133
Long Philtrum	0.0% (0/26)	17.5% (22/126)	15.4% (2/13)	16.7% (20/120)	2.654	0.448	0.136
Cleft Lip	0.0% (0/28)	16.9% (24/142)	9.1% (1/11)	7.1% (9/126)	4.824	0.185	0.184
Single Atrium	10.7% (3/28)	11.3% (17/150)	0.0% (0/11)	10.9% (14/128)	0.863	0.834	0.709
Low Birth Weight	0.0% (0/12)	3.6% (2/54)	0.0% (0/2)	18.6% (8/43)	4.054	0.256	0.272
Postnatal Microcephaly	0.0% (0/18)	0.0% (0/52)	0.0% (0/2)	18.2% (6/33)	7.163	0.067	0.364
Developmental Delay	10.0% (2/20)	4.9% (4/82)	50.0% (2/4)	14.0% (6/43)	5.357	0.147	0.269
Prenatal Microcephaly	0.0% (0/10)	4.3% (2/46)	0.0% (0/2)	14.6% (6/41)	2.066	0.559	0.208
Pectus Carinatum	7.7% (2/26)	10.9.% (10/92)	0.0% (0/2)	4.8% (4/83)	1.220	0.748	0.108
Clinodactyly	0.0% (0/24)	1.3% (2/152)	0.0% (0/15)	15.6% (20/128)	12.800	**0.005**	0.282
Hypertelorism	0.0% (0/26)	0.0% (0/126)	0.0% (0/13)	15.0% (18/120)	13.290	**0.004**	0.304
Club Foot	3.6% (1/28)	4.7% (7/148)	30.8% (4/13)	1.8% (2/110)	8.996	**0.029**	0.271

[a] Phenotype frequency is represented by the absolute number (N) for the patients in which the phenotype was assessed (Total), with the respective proportion (%).
[b] An adjusted Chi-square test was used to account for the virtual duplication of sample size.
[c] Significance values in bold mean that $p < 0.050$.

Supplementary Table 11. General phenotypes (by system) in patients with variants affecting both *EVC* and *CRMP1* versus *EVC* only.

Feature[a]	***EVC* Only %(N/Total)**	***EVC* & *CRMP1* %(N/Total)**	Adjusted Chi-square[b]	Adjusted Sig. (p)[c]	Adjusted Cramer's V
Skeletal Anomaly	96.1% (49/51)	100% (157/157)	3.068	0.080	0.173
Skeletal Anomaly (excluding polydactyly)	80.4% (41/51)	88.2% (135/153)	0.942	0.332	0.099
Facial Feature	87.5% (42/48)	80.0% (104/130)	0.669	0.818	0.087
Congenital Heart Disease	62.7% (32/51)	66.1% (84/127)	0.135	0.714	0.032
Thoracic Anomaly	36.0% (18/50)	55.9% (76/136)	2.891	0.089	0.176

[a] Phenotype frequency is represented by the absolute number (N) for the patients in which the phenotype was assessed (Total), with the respective proportion (%)
[b] An adjusted Chi-square test was used to account for the virtual duplication of sample size. Note that neurological and genital phenotypes were not included as there were no cases in at least one of the categories of variants.
[c] Significance values in bold mean that $p < 0.050$.

Supplementary Table 12. Specific phenotypes in patients with variants affecting both *EVC* and *CRMP1* versus *EVC* only.

Feature[a]	***EVC* Only %(N/Total)**	***EVC* & *CRMP1* %(N/Total)**	Adjusted Chi-square[b]	Adjusted Sig. (p)[c]	Cramer's V
Postaxial Polydactyly (Hand)	92.2% (47/51)	100% (157/157)	6.195	**0.013**	0.246
Bilateral	100% (47/47)	98.7% (155/157)	0.307	0.580	0.054
Short Stature	66.7% (24/36)	78.8% (52/66)	0.901	0.343	0.133
Limb Shortening	64.7% (33/51)	79.6% (117/147)	2.171	0.141	0.152
Nail Dysplasia/Hypoplasia	72.3% (34/47)	67.2% (84/125)	0.138	0.710	0.049
Hypodontia	59.0% (23/39)	65.3% (49/75)	0.191	0.663	0.063
Alveolar Ridge Defect	51.2% (21/41)	56.0% (61/109)	0.098	0.755	0.042
Atrial Septal Defect	45.1% (23/51)	60.6% (77/127)	1.646	0.200	0.142
Narrow Chest	32.0% (16/50)	55.9% (76/136)	4.171	**0.041**	0.212
Short Ribs	32.0% (16/50)	55.9% (76/136)	4.171	**0.041**	0.212
Short and Thickened Tubular Bones	23.9% (11/46)	47.1% (49/104)	4.342	**0.037**	0.218
Delayed Eruption of Teeth	48.6% (17/35)	52.4% (33/63)	0.045	0.832	0.037
Other Dysmorphisms	32.6% (15/46)	31.3% (35/112)	0.024	0.877	0.013
Other Congenital Heart Defects	30.0% (15/50)	42.7% (53/124)	1.127	0.288	0.118
Limb Shortening at Birth	7.7% (2/26)	46.9% (30/64)	6.194	**0.013**	0.371
Low Iliac Wings	11.1% (5/45)	33.3% (31/93)	4.839	**0.028**	0.237
Low Weight	6.5% (2/31)	26.7% (12/45)	2.724	0.099	0.256
Acetabula Spur Projections	8.9% (4/45)	28.0% (26/93)	3.450	0.063	0.217
Postaxial Polydactyly (Foot)	29.4% (25/51)	21.4% (31/145)	0.818	0.366	0.083
Bilateral	80.0% (12/15)	77.4% (24/31)	<0.001	0.999	0.029
Pre-Natal Limb Shortening	19.4% (6/31)	28.6% (14/49)	0.453	0.501	0.104
Upper-Lip Defect	6.7% (3/45)	36.6% (45/123)	8.431	**0.004**	0.293
Genu Valgum	20.4% (10/49)	19.8% (24/121)	0.001	0.972	0.006
Abnormal Birth Stature	7.7% (2/26)	22.7% (10/44)	1.300	0.254	0.193
Brachydactyly	12.8% (6/47)	21.7% (28/129)	0.927	0.336	0.100
Neonatal Teeth	5.9% (2/34)	33.3% (24/72)	4.700	**0.030**	0.298

Short Broad Nose	6.8% (3/44)	21.3% (23/108)	1.896	0.169	0.174
Ventricular Septal Defect	14.0% (7/50)	21.6% (25/116)	0.499	0.480	0.088
Cone-Shaped Epiphyses of Phalanges	22.2% (10/45)	19.0% (20/105)	0.083	0.773	0.036
Syndactyly	19.1% (9/47)	10.1% (13/129)	1.522	0.217	0.121
Capitate-Hamate Fusion	13.3% (6/45)	23.3% (24/103)	1.003	0.317	0.114
Long Philtrum	4.5% (2/44)	18.5% (20/108)	2.466	0.116	0.180
Cleft Lip	0.0% (0/46)	19.4% (24/124)	5.183	**0.023**	0.247
Single Atrium	9.8% (5/51)	11.8% (15/127)	0.016	0.900	0.029
Low Birth Weight	0.0% (0/28)	5.0% (2/40)	0.721	0.396	0.146
Postnatal Microcephaly	0.0% (0/29)	0.0% (0/41)	NA	NA	NA
Developmental Delay	18.2% (6/33)	0.0% (0/69)	6.375	**0.012**	0.361
Prenatal Microcephaly	7.7% (2/26)	0.0% (0/30)	1.197	0.274	0.207
Pectus Carinatum	0.0% (0/38)	15.0% (12/80)	1.781	0.075	0.232
Clinodactyly	0.0% (0/47)	1.6% (2/129)	0.373	0.541	0.065
Hypertelorism	0.0% (0/44)	0.0% (0/108)	NA	NA	NA
Club Foot	10.0% (5/50)	2.4% (3/126)	2.494	0.114	0.165

[a] Phenotype frequency is represented by the absolute number (N) for the patients in which the phenotype was assessed (Total), with the respective proportion (%).
[b] An adjusted Chi-square test was used to account for the virtual duplication of sample size.
[c] Significance values in bold mean that $p < 0.050$.

Supplementary Table 13. General phenotypes (by system) in patients with two variants affecting *EVC* only, one variant affecting *EVC* only and another affecting *CRMP1*, or two variants affecting *CRMP1*.

Feature[a]	**Both Variants on *EVC* Only % (N/Total)**	**One *EVC* and One *CRMP1* Variant % (N/Total)**	**Both Variants on *CRMP1* % (N/Total)**	Chi-square	Sig. (p)[b]	Cramer's V
Skeletal Anomaly	94.4% (17/18)	100.0% (15/15)	100.0% (71/71)	4.824	0.090	0.215
Skeletal Anomaly (excluding polydactyly)	77.8% (14/18)	86.7% (13/15)	88.4% (61/69)	1.364	0.506	0.116
Facial Feature	88.2% (15/17)	85.7% (12/14)	79.3% (46/58)	0.864	0.649	0.099
Congenital Heart Disease	50.0% (9/18)	93.3% (14/15)	62.5% (35/56)	7.242	**0.027**	0.285
Thoracic Anomaly	38.9% (7/18)	28.6% (4/14)	59.0% (36/61)	5.434	0.066	0.242
Neurological Disease	20.0% (3/15)	0.0% (0/10)	0.0% (0/49)	12.299	**0.002**	0.408
Genital Anomaly	9.1% (1/11)	12.5% (1/8)	7.4% (2/27)	0.204	0.903	0.067

[a] Phenotype frequency is represented by the absolute number (N) for the patients in which the phenotype was assessed (Total), with the respective proportion (%).
[b] Significance values in bold mean that $p < 0.050$.

Supplementary Table 14. Specific phenotypes in patients with two variants affecting *EVC* only, one variant affecting *EVC* only and another affecting *CRMP1*, or two variants affecting *CRMP1*.

Feature[a]	**Both Variants on *EVC* Only % (N/Total)**	**One *EVC* and One *CRMP1* Variant % (N/Total)**	**Both Variants on *CRMP1* % (N/Total)**	Chi-square	Sig. (p)[b]	Cramer's V
Postaxial Polydactyly (Hand)	88.9% (16/18)	100.0% (15/15)	100.0% (71/71)	9.743	**0.008**	0.306
Bilateral	100.0% (16/16)	100.0% (15/15)	98.6% (70/71)	0.441	0.802	0.066
Short Stature	62.5% (10/16)	100.0% (4/4)	77.4% (24/31)	2.722	0.256	0.231
Limb Shortening	61.1% (11/18)	73.3% (11/15)	80.3% (53/66)	2.893	0.235	0.171
Nail Dysplasia/ Hypoplasia	82.4% (14/17)	46.2% (6/13)	69.6% (39/56)	4.562	0.102	0.230
Hypodontia	56.3% (9/16)	71.4% (5/7)	64.7% (22/34)	0.569	0.752	0.100
Alveolar Ridge Defect	56.3% (9/16)	33.3% (3/9)	58.0% (29/50)	1.893	0.388	0.159
Atrial Septal Defect	33.3% (6/18)	73.3% (11/15)	58.9% (33/56)	5.781	0.056	0.255
Narrow Chest	33.3% (6/18)	28.6% (4/14)	59.0% (36/61)	6.545	**0.038**	0.265
Short Ribs	33.3% (6/18)	28.6% (4/14)	57.6% (34/59)	5.825	0.054	0.254
Short and Thickened Tubular Bones	12.5% (2/16)	50.0% (7/14)	46.7% (21/45)	6.458	**0.040**	0.293
Delayed Eruption of Teeth	42.9% (6/14)	71.4% (5/7)	50.0% (14/28)	1.552	0.460	0.178
Other Dysmorphisms	25.0% (4/16)	50.0% (7/14)	28.6% (14/49)	2.721	0.257	0.186
Other Congenital Heart Defects	22.2% (4/18)	50.0% (7/14)	41.8% (23/55)	3.023	0.221	0.186
Limb Shortening at Birth	10.0% (1/10)	0.0% (0/6)	51.7% (15/29)	9.469	**0.009**	0.459
Low Iliac Wings	0.0% (0/16)	38.5% (5/13)	32.5% (13/40)	7.533	**0.023**	0.330
Low Weight	7.1% (1/14)	0.0% (0/3)	28.6% (6/21)	3.302	0.192	0.295
Acetabula Spur Projections	0.0% (0/16)	30.8% (4/13)	27.5% (11/40)	5.848	0.054	0.291
Postaxial Polydactyly (Foot)	27.8% (5/18)	33.3% (5/15)	20.0% (13/65)	1.434	0.488	0.121
Bilateral	100.0% (5/5)	40.0% (2/5)	84.6% (11/13)	6.000	0.050	0.511
Pre-Natal Limb Shortening	18.2% (2/11)	22.2% (2/9)	30.0% (6/20)	0.576	0.750	0.120
Upper-Lip Defect	0.0% (0/17)	27.3% (3/11)	37.5% (21/56)	8.997	**0.011**	0.327

Genu Valgum	22.2% (4/18)	15.4% (2/13)	20.4% (11/54)	0.233	0.890	0.052
Abnormal Birth Stature	10.0% (1/10)	0.0% (0/6)	26.3% (5/19)	2.726	0.256	0.279
Brachydactyly	6.3% (1/16)	26.7% (4/15)	21.1% (12/57)	2.383	0.304	0.165
Neonatal Teeth	7.1% (1/14)	0.0% (0/6)	36.4% (12/33)	6.733	**0.035**	0.356
Short Broad Nose	0.0% (0/16)	25.0% (3/12)	20.8% (10/48)	4.300	0.117	0.238
Ventricular Septal Defect	11.1% (2/18)	21.4% (3/14)	21.6% (11/51)	0.985	0.611	0.109
Cone-Shaped Epiphyses of Phalanges	25.0% (4/16)	15.4% (2/13)	19.6% (9/46)	0.429	0.807	0.076
Syndactyly	25.0% (4/16)	6.7% (1/15)	10.5% (6/57)	2.955	0.228	0.183
Capitate-Hamate Fusion	18.8% (3/16)	0.0% (0/13)	26.7% (12/45)	4.467	0.107	0.246
Long Philtrum	0.0% (0/16)	16.7% (2/12)	18.8% (9/48)	3.463	0.177	0.213
Cleft Lip	0.0% (0/17)	0.0% (0/12)	21.4% (12/56)	7.236	**0.027**	0.292
Single Atrium	5.6% (1/18)	20.0% (3/15)	10.7% (6/56)	1.753	0.416	0.140
Low Birth Weight	0.0% (0/11)	0.0% (0/6)	5.9% (1/17)	1.030	0.597	0.174
Postnatal Microcephaly	0.0% (0/13)	0.0% (0/3)	0.0% (0/19)	NA	NA	NA
Developmental Delay	21.4% (3/14)	0.0% (0/5)	0.0% (0/32)	8.424	**0.015**	0.406
Prenatal Microcephaly	10.0% (1/10)	0.0% (0/6)	0.0% (0/12)	1.867	0.393	0.258
Pectus Carinatum	0.0% (0/10)	0.0% (0/6)	16.2% (6/37)	3.971	0.137	0.259
Clinodactyly	0.0% (0/16)	0.0% (0/15)	1.8% (1/57)	0.550	0.760	0.079
Hypertelorism	0.0% (0/16)	0.0% (0/12)	0.0% (0/48)	NA	NA	NA
Club Foot	5.6% (1/18)	21.4% (3/14)	0.0% (0/56)	11.906	**0.003**	0.368

[a] Phenotype frequency is represented by the absolute number (N) for the patients in which the phenotype was assessed (Total), with the respective proportion (%).
[b] Significance values in bold mean that $p < 0.050$. NA, not applicable.

Systematic Review of Ellis-van Creveld Case Reports/Series – Protocol

This protocol was adapted from the COSMOS-E guidelines on conducting systematic reviews and meta-analyses of observational studies of etiology.

Research Question

To compile and review all reported cases of disease-causing variants in the *EVC* or *EVC2* genes, associated with Ellis-van Creveld syndrome and Weyers acrofacial dysostosis.

Inclusion Criteria

Original case reports/series of patients with likely pathogenic or pathogenic variants in the *EVC* or *EVC2* gene (heterozygous, compound heterozygous or homozygous). Cases can occur in the same family or not.

Exclusion Criteria

Patients with any other chondroectodermal dysplasia/ciliopathy without any identified likely pathogenic or pathogenic variant in the *EVC* or *EVC2* gene after diagnostic assessment of both SNVs and CNVs.

Databases for the Search

MEDLINE (PubMed), ClinVar.

Terms for the Search in MEDLINE

Ellis-van Creveld Syndrome [MeSH Term] OR
EVC protein, human [MeSH Term] OR
EVC2 protein, human [MeSH Term] OR
EVC gene AND human OR
EVC2 gene AND human OR
Weyers acrofacial dysostosis [MeSH Term] OR
Weyers acrodental dysostosis [MeSH Term].

Terms for the Search in ClinVar

EVC - HGNC:3497

EVC2 - HGNC:19747

Query for MEDLINE

(Ellis-van Creveld Syndrome) OR (EVC protein, human) OR (EVC2 protein, human) OR ((EVC gene) AND (human)) OR ((EVC2 gene) AND (human)) OR (Weyers acrofacial dysostosis) OR (Weyers acrodental dysostosis)
